# A dual-path framework for enhancing student engagement and learning outcomes in sports education: Integrating technology acceptance, self-regulation, and self-efficacy

**DOI:** 10.1371/journal.pone.0345809

**Published:** 2026-03-25

**Authors:** Guifeng Zheng, Yanting Wang, Juan Du

**Affiliations:** 1 Physical Education Department, Xiamen University Tan Kah Kee College, Zhangzhou, China; 2 College of Physical Education, Quanzhou Normal University, Quanzhou, China; 3 Department of Public Physical Education, Fujian Agriculture and Forestry University, Fuzhou, China; Hunan Normal University, CHINA

## Abstract

The rapid adoption of mobile learning technologies in education has presented new opportunities and challenges for the innovative transformation of physical education. This study expands the Technology Acceptance Model (TAM) to systematically analyze how mobile applications influence learning behaviors in higher education physical education. Specifically, it explores the role of mobile technology in hybrid physical education environments by integrating the theoretical dimensions of self-regulated learning, learning self-efficacy, and technology acceptance. Findings reveal that students’ perceptions of usefulness and attitudes toward usage significantly and positively impact their behavioral intentions and self-regulated learning abilities, while also indirectly enhancing learning self-efficacy. This theoretical extension not only provides valuable insights into the mechanisms driving technology-based educational innovation but also introduces a new analytical framework for hybrid physical education. The findings have important practical implications for technology integration and learning behavior optimization in higher education.

## 1. Introduction

With the rapid evolution of digital technology, educational practices are undergoing profound changes. Mobile learning has emerged as a key development direction in contemporary education, offering support for learning beyond traditional classrooms through its high mobility, contextual adaptability, and real-time interactivity. Traditional teaching methods often face challenges such as limited student engagement and uneven resource distribution in the field of physical education. Mobile learning technology holds promise to enrich the teaching ecosystem by delivering instantaneous, interactive, and personalized learning experiences, thereby promoting the development of motor skills and enhancing physical literacy.

The Technology Acceptance Model (TAM) is a theoretical framework used to explain user adoption of technology. Although the TAM has been widely used to study is a commonly used to study technology adoption, its traditional form often emphasizes instrumental variables such as perceived usefulness (PU) and perceived ease of use (PEOU), while treating the learning process as a secondary factor. For physical education (PE), students’ motivational states, self-regulation abilities, and self-efficacy beliefs play a crucial role in translating technology use into meaningful learning outcomes [1,2]. Recent literature indicates that technology adoption does not automatically translate into sustained engagement or long-term behavioral change, making it crucial to integrate instructional design with learners’ psychological mechanisms [3,4]. This study addresses this gap by integrating Self-Regulated Learning (SRL) and Learning Self-Efficacy (LSE) into the TAM framework, thereby better explaining how mobile physical education applications influence student engagement and learning outcomes.

The study introduces a dual-path framework — the Technology Acceptance Path and the Psychological Regulation Path — integrating perceived usefulness (PU), perceived ease of use (PEOU), self-regulated learning (SRL), and learning self-efficacy (LSE) to predict usage intention and learning outcomes in physical education (PE) mobile learning. This study examines the following questions: (1) How do PU and PEOU influence willingness to use through the mediating effects of SRL and LSE? (2) Do SRL and LSE interact to enhance learning engagement and learning outcomes? (3) What instructional design implications emerge for optimizing app features to promote self-directed learning and technological efficacy? Theoretically, this study incorporates SRL and LSE as key psychological mechanisms into the TAM, thereby extending TAM to provide a more robust explanation for sustained engagement and enhanced learning outcomes in mobile physical education. Practically, the findings will guide the development of physical education applications and interventions, aligning interface design, feedback, and goal-setting with students’ self-regulation and self-efficacy to foster autonomous learning and elevate students’ perceived technological efficacy.

## 2. Literature review

Blended physical education, integrating digital resources with traditional methods, has become increasingly important for enhancing student engagement and learning outcomes. This study investigates how campus fitness running apps, such as Chuanggao, affect students’ technology acceptance in physical education, using a framework based on the TAM and incorporating SRL and LSE. The theoretical limitations and psychological mechanisms of the TAM model are examined, along with the transformation and challenges of mobile learning technology in physical education and the relationship between LSE and SRL.

### 2.1. Mobile learning technology in physical education: Transformations and challenges

The rapid advancement of digital technology is reshaping the landscape of physical education instruction [5,6]. As a key driver of educational innovation, mobile learning technology enables real-time feedback, contextualized learning, and personalized training, effectively breaking through the constraints of time and space inherent in traditional teaching methods [7,8].

However, research indicates that merely introducing technology does not guarantee sustained learning motivation or continuous skill development in physical education courses [9–11]. Existing research predominantly focuses on the performance metrics of the technological tools themselves (e.g., sensor accuracy, feedback latency), while relatively neglecting psychological factors such as learners’ motivational states, self-regulation abilities, and self-efficacy [12,13]. Particularly during the COVID-19 pandemic, sports-related applications became crucial tools for universities to maintain physical education instruction. These applications effectively reinforce students’ intrinsic motivation and explicit exercise habits [14–16] while providing educators with innovative teaching resources and methodologies. Advances in artificial intelligence have further optimized application functionality. The introduction of intelligent training recommendations, motion recognition, and error correction capabilities enhances the scientific rigor and adaptability of fitness apps, improving learning engagement and outcomes by creating immersive exercise experiences.

Fitness applications should not be confined to behavioral monitoring tools [17] but should also promote students’ active learning and physical and mental development [18,19]. How to achieve an organic integration of technology, instructional design, and learner psychology remains a core research topic [20–23]. We need a more in-depth understanding of how instructional design, technology application, and learner psychology interact to jointly influence engagement and learning outcomes in physical education settings.

### 2.2. Theoretical boundaries and psychological mechanisms of the technology acceptance model

TAM as a common framework for understanding technology adoption, emphasizes perceived usefulness (PU) and perceived ease of use (PEOU) as predictors of intention to use. However, this instrumental perspective limits its explanatory power regarding the learning process in physical education [2,24], making it even more challenging to fully account for the complex psychological mechanisms underlying technology use [25].

Recent studies on the traditional TAM indicate that merely accepting technology does not guarantee long-term behavioral change. Integrating psychological constructs such as self-regulated learning (SRL) and learning self-efficacy (LSE) is necessary to gain deeper insights into the intrinsic motivations driving technology use [4,26]. Zimmerman (2000) [27] proposed the theory of self-regulated learning, emphasizing that learning is an active process where students proactively regulate their cognitive, motivational, and behavioral strategies. Navarro et al.(2023) [28] further confirmed that students’ self-efficacy and self-regulation abilities are key factors in their technology adoption. Thus, the impact of mobile learning technology on student learning behaviors largely depends on their self-regulation skills and confidence in learning abilities [15]. This finding challenges the linear assumptions of traditional technology acceptance models, highlighting the importance of complex psychological factors. Traditional physical education faces issues such as low participation, insufficient motivation, and monotonous practice methods. Sports applications like Chuanggao App, however, stimulate students’ intrinsic learning motivation while providing technological tools. Students’ acceptance of sports management systems and perceived usefulness (PU) exert a significant positive influence on behavioral intention (BI). Perceived ease of use (PEOU) indirectly affects behavioral intention (BI) through PU, thereby validating the mediating role of ATM [29–32]. Yardley et al.(2016) [33] argue that technological availability alone cannot guarantee sustained learning engagement and behavioral change. Educational mobile technology involves complex interactions between technological and psychological factors, far from being a simple tool substitution. Therefore, integrating SRL and LSE with TAM provides a more comprehensive explanation of how physical education technology influences student engagement and learning outcomes through cognitive, motivational, and behavioral pathways.

### 2.3. Synergy between SRL and LSE and their educational value

Self-regulated learning (SRL) underscores learners’ autonomy in setting goals, adjusting strategies, and monitoring progress [34], while learning self-efficacy (LSE) refers to individuals’ belief in their ability to complete learning tasks [35]. Research indicates that the synergistic effect of SRL and LSE significantly enhances students’ mobile learning outcomes [36]. In physical education (PE) applications, sports apps like Chuanggao can indirectly enhance students’ SRL by reinforcing goal-oriented behaviors(e.g., creating personalized training plans). Additionally, students with higher LSE are more likely to perceive such apps as empowerment tools for promoting physical health [37].

Mobile learning in sports is driving an evolution from basic information delivery to comprehensive skill development, yet it still faces complex challenges. Traditional Technology Acceptance Model (TAM) struggle to explain the interplay between technology, psychology factors, and learning behaviors, promoting researchers to explore psychological constructs like SRL and LSE. Current research exhibits two major limitations: (1) Most studies focus solely on the isolated effects of sports app instruction on SRL or LSE, neglecting their interactions; (2) Integrative analyses grounded in the TAM framework remain nascent, with unclear relationships between relevant psychological variables and technology acceptance. Although evidence suggests both SRL and LSE correlate with technology use, research on their interactions within the TAM framework, particular in physical education remains limited. Consequently, reassessing the compatibility between technological tools and learning psychology becomes crucial.

**Fig 1:**
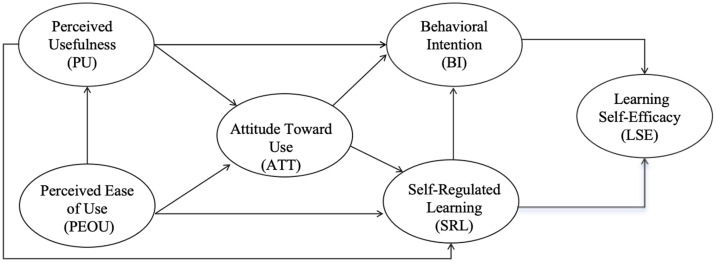
Conceptual Model. Note: The model incorporates six key constructs: Perceived Usefulness (PU), Perceived Ease of Use (PEOU), Attitude Towards Use (ATT), Self-Regulation Learning (SRL), Behavioral Intention (BI), and Learning Self-Efficacy (LSE).

Consequently, we propose integrating SRL (self-regulated learning) and LSE (learning self-efficacy) into the TAM (Technology Acceptance Model)(see Fig 1). This framework illustrates the core constructs and their hypothesized relationships within the context of a campus running fitness application. Key variables include Perceived Usefulness (PU), Perceived Ease of Use (PEOU), Behavioral Intention (BI) to use the app, Self-Regulated Learning (SRL), and Learning Self-Efficacy (LSE). The model posits that PU and PEOU directly influence BI, while SRL and LSE act as antecedent psychological factors that interact with these technology perceptions to shape student acceptance. Specifically, SRL is proposed to indirectly enhance usage intention by strengthening goal-oriented behaviors, such as formulating personalized training plans, whereas students with higher LSE are more likely to perceive the fitness app as an empowering tool. This aims to explore whether SRL and LSE can jointly predict the intention to use and learning outcomes driven by perceived usefulness/ perceived ease of use (PU/PEOU), and how their synergistic effects can guide the design of mobile physical education applications.

## 3. Methodology

### 3.1. Context

The study was approved by the ethics committee of the second author’s institution (Approval Number: QZSYLL2024032) and conducted at a public university in southeastern China on 21/07/2024. Prior to distributing questionnaires, the research team obtained oral informed consent from all adult participants, detailing the research purpose, significance, and intended use. Regarding informed consent, participants stated that filling out the questionnaire represented their agreement to take part in the survey following a thorough oral explanation of the research’s contents by the research team (including data processing, privacy protection, potential risks and benefits, and the principle of voluntary participation). This study used anonymized data to protect participant privacy. Integrating educational psychology and technology acceptance theory, it moves beyond traditional TAM limitations, offering a new theoretical framework for blended physical education and empirical insights for app developers to enhance feature design.

### 3.2. Subject

This study focuses on selecting students with experience in using mobile technologies to efficiently and accurately gather high-quality data. The goal is to provide robust empirical support for developing a technology-psychology dual-path model while minimizing research expenses. To achieve this, a convenience sampling method was employed, administering a questionnaire survey to students enrolled in physical education courses at XXX University during the 2023–2024 academic year, specifically from 21/09/2024–23/09/2024. The sample covers four grade levels, from freshmen to seniors years. The study requires all participants to complete at least two semesters of the Chuanggao App extracurricular running task to ensure a consistent experience in using the App. The research team distributed 300 questionnaires, of which 289 valid responses were obtained, resulting in an effective recovery rate of 96.3%. The sample encompassed different disciplines and academic levels, which are representative to a certain extent. Demographic characteristics are detailed in Table 1.

**Table 1:** Demographic background of participants (N = 289).

Category	Project	Number	Percentage
Gender	Male	152	52.6%
	Female	137	47.4%
Grade	Freshman	78	27.0%
	Sophomore	85	29.4%
	Junior	72	24.9%
	Senior	54	18.7%
Subject background	Liberal arts	123	42.6%
	Science and engineering	113	39.1%
	Agriculture and forestry	53	18.3%

### 3.3. Research design

This cross-sectional study explored the psychological mechanisms driving the adoption of sports APP technology by integrating the Chuanggao App into a four-semester physical education curriculum that combined classroom instruction with extracurricular activities.Intervention structure consisted of:

- In-class sessions: 90 minutes of weekly physical activity and skill-based instruction.
- Extracurricular sessions: Mandatory running exercises monitored through the APP utilizing GPS tracking.

Grading Protocol

- Baseline: 15 runs/semester (60 points)
- Proficiency: 36 + runs/semester (100 points)
- Performance Metrics:
- Step rate ≥ 50 steps/minute
- Pace: 3–9 minutes/km
- Duration ≤ 25 minutes/session

Temporal Framework:

- Pre-test: APP training (Weeks 1–2)
- Implementation: Weeks 3–18
- Post-test: Learning efficacy assessment (Week 19)

### 3.4. Data collection and questionnaire design

The study participants were university students who had completed extracurricular physical exercise for at least two semesters using the Chuanggao App. At the end of the 2023–2024 semester, their learning outcomes would be assessed based on their grades in physical activity course. Participants also completed the Questionnaire on College Students’ Willingness to Use Chuanggao App for Physical Activity Courses and Learning Effects, after which their course performance was analyzed.

This study employed convenience sampling, which, while limited in terms of structural generalizability, sample representativeness, and external validity, was chosen due to its focus on identifying psychological mechanisms of technology acceptance and conducting conceptual validation rather than statistical inference. The selected sample was easily accessible and homogeneous. This methodology provides an initial theoretical framework for educational technology research and lays the groundwork for subsequent in-depth studies, thereby holding academic value. Thus, the research team distributed 300 questionnaires to eligible students and collected responses on-site in early July 2024 (semester end), and the invalid questionnaires were excluded based on the following criteria:

- More than 20% of questions unanswered
- Patterned responses (e.g., selecting the same option for 10 consecutive questions)
- Completion time was within 2 minutes (the reasonable range determined by pilot testing was 5–8 minutes)

A total of 11 invalid questionnaires were discarded. Prior to the formal survey, a pilot test was conducted with 30 randomly selected students. The results demonstrated high reliability, with a Cronbach’s α > .80.

### 3.5. Measurement

(1) Evaluation of learning achievements

Learning performance was evaluated based on the average effective exercise scores of students in four semesters of the physical activity program. These scores were determined by the teacher through a system that assesses whether the exercise meets the following three criteria simultaneously: a stride frequency of ≥50 steps/ minute, a pace range 3–9 minutes/ km, and a single exercise duration of ≤25 minutes.

Scoring criteria are as follows: 60 points for 15 valid exercises, and 100 points for completing ≥36 valid exercises. The corresponding scores based on exercise completion times are shown in Table 2.

**Table 2:** Scoring table for effective number for long-distance runs per semesters.

Valid Times (times)	0	1-14	15	16-17	18-19	20-21	22-24	25-27	28-30	31-35	36
Semester Score (points)	0	50	60	65	70	75	80	85	90	95	100

**Note:** Scoring criteria for long-distance running frequency. Participants received 0–100 points based on the number of completed runs per semester (0 runs = 0 points; 36 + runs = 100 points).

(2) College students ‘willingness to use Chuanggao App questionnaire

The questionnaire design includes six dimensions: perceived ease of use, perceived usefulness, attitude towards use, behavior intention, self-regulated learning and learning self-efficacy. Given that the original model by Davis et al.(1989) [38] struggles to effectively address the complex technological acceptance mechanisms of mobile applications and their inherent contextual limitations within modern blended learning environments, particularly in physical education, we have adopted the Technology Acceptance Model (TAM) proposed by Moreno et al.(2017) [39]. This approach aims to more accurately understand and capture the technology acceptance characteristics of mobile applications in physical education. The scale covers four dimensions: perceived usefulness (4 items), perceived ease of use (3 items), attitude to use (3 items), and behavior intention (3 items). Additionally, self-regulated learning (SRL) was evaluated using the scale developed by Wang & Tsai (2016) [40], which consists of 6 items, and learning self-efficacy (LSE) adopted the scale by Lu et al.(2017) [41], which contains 5 items. All items were rated on a 5-point Likert scale (1 = “strongly disagree” to 5=”strongly agree”) and reviewed by a bilingual panel expert to ensure semantic coherence.

### 3.6. Data analysis and model fit

Statistical analysis was conducted using Partial Least Squares Structural Equation Modeling (PLS-SEM), a method selected for its suitability with smaller sample sizes (n ≥ 100) and its relaxed assumptions regarding normal distribution [42], which is suitable for analysis of 289 samples in this study. Furthermore, PLS-SEM is particularly effective for estimating path coefficients and testing hypotheses involving multiple mediating variables [43]. Stone-Geisser’s Q² was employed to evaluate the predictive ability of the model, which is consistent with the goal of this study to examine the relationship between technology acceptance and learning effectiveness.

The details results of the study are presented in Tables 3–5, as follows: the model fit test indicated that the Cronbach’s α coefficients within each dimension had a high internal consistency (.83 −.86) and a good combined reliability (CR:.77 −.93), all exceeding the.70 threshold. Convergent validity was measured by the average variance extracted (AVE), which ranged from.53 to.82, meeting the criterion of ≥.50. Discriminant validity was confirmed that the square root of AVE exceeded the correlation coefficients with other dimensions, and HTMT values were below.90. Regarding the overall fitness model, the standardized root mean square residual (SRMR = .062) and the normed fit index (NFI = .91), were better than the critical values of.08 and.90, respectively, further indicating that the model had a good model fit [44].

**Table 3:** Indicators for Measurement and Assessment of Model Fit Quality.

Construct	Factor loadings	*Mean* (*SD*)	VIF	Common Method Bias
Perceived Usefulness (PU)				0.036
Cronbach's α = 0.84, C.R. = 0.81, AVE = 0.58				
PU1:The chuanggao app could help me motivate and solve my reluctance to run.	0.76	3.66(0.91)	1.45	
PU2: The chuanggao app inspires me to run regularly, aiding in building a consistent exercise routine.	0.79	3.56(0.94)	1.52	
PU3: The Chuanggao app proves to be quite beneficial for me.	0.74	3.67(0.91)	1.62	
Perceived Ease of Use (PEOU)				0.042
Cronbach's α = 0.83, C.R. = 0.92, AVE = 0.73				
PEOU1: The chuanggao APP is easy to use.	0.79	3.65(0.95)	1.48	
PEOU2:The content of chuanggao app is clear and easy to understand.	0.93	3.81(0.84)	1.75	
PEOU3: I think using the Chuanggao app or browsing its content and information doesn't take up much time.	0.94	3.75(0.87)	1.72	
PEOU4: The app enables users to monitor their pace, track workout counts, and view school rankings anytime, encouraging them to stay motivated and enhance their exercise routines.	0.75	3.57(0.91)	1.55	
Attitude Towards Use (ATT)				0.029
Cronbach's α = 0.86, C.R. = 0.77, AVE = 0.53				
ATT1: I find this app quite enjoyable to use.	0.72	3.48(0.93)	1.52	
ATT2: I would continue using this app if it receives regular updates with new content and software enhancements.	0.72	3.60(0.91)	1.71	
ATT3: I believe it is necessary to use this app to help develop good exercise habits.	0.74	3.61(0.89)	1.63	
Self-Regulation Learning (SRL)				0.033
Cronbach's α = 0.85, C.R. = 0.93, AVE = 0.82				
SRL1: I tailor my speed and activity range based on the pace and motion range suggestions offered by the system when using this app.	0.94	3.71(0.88)	1.59	
SRL2: I will evaluate whether it has enhanced my performance in middle- and long-distance running, after using this app for either a semester of training or just one session.	0.94	3.69(0.88)	1.79	
SRL3: I will reflect on whether maintaining consistent exercise over four semesters has successfully helped me build a lasting habit of physical activity, after using this app for either a semester of regular workouts or just one session.	0.84	3.66(0.89)	1.69	
Behavioral Intention (BI)				0.042
Cronbach's α = 0.85, C.R. = 0.82, AVE = 0.60				
BI1: I enjoy using the Chuanggao app.	0.78	3.41(0.89)	1.55	
BI2: I would recommend this app to others.	0.76	3.44(0.83)	1.68	
BI3: I am thoroughly pleased with the overall user experience of this app.	0.78	3.56(0.93)	1.57	
Learning Self-Efficacy (LSE)				0.025
Cronbach's α = 0.86, C.R. = 0.85, AVE = 0.58				
LSE1: Using this app helps me regulate my speed and breathing with pace reminders, allowing me to build a consistent exercise routine.	0.75	3.51(0.95)	1.68	
LSE2: Using this app taught me how to synchronize my cadence with my breathing, which helped me enhance my pace.	0.74	3.54(0.97)	1.88	
LSE3: Using this app, I enhanced my endurance running performance in fitness tests by leveraging its intelligent features, such as managing running pace, tracking individual run distances, and monitoring total running distance.	0.79	3.61(0.95)	1.75	
LSE4: Using this app taught me how to effectively integrate fragmented time with extracurricular activities, significantly enhancing my time management skills.	0.76	3.60(0.93)	1.72	
Goodness of Fit				
SRMR	0.062			
NFI	0.91			
Q²	0.48			

**Table 4:** Fornell–Larcker Discriminant Validity Matrix.

Construct	PU	PEOU	ATT	SRL	BI	LSE	AVE	sqrt(AVE)
PU	0.76	0.42	0.38	0.55	0.50	0.44	0.58	0.76
PEOU		0.85	0.41	0.33	0.28	0.31	0.73	0.85
ATT			0.73	0.46	0.40	0.39	0.53	0.73
SRL				0.91	0.55	0.62	0.82	0.91
BI					0.77	0.58	0.60	0.77
LSE						0.76	0.58	0.76

**Note.** AVE: Average Variance Extracted.

**Table 5:** HTMT Discriminant Validity Matrix (Upper triangle).

Construct	PU	PEOU	ATT	SRL	BI	LSE
PU	1.00	0.68	0.57	0.42	0.49	0.53
PEOU		1.00	0.49	0.41	0.37	0.39
ATT			1.00	0.46	0.40	0.39
SRL				1.00	0.55	0.62
BI					1.00	0.58
LSE						1.00

**Note.** HTMT: Heterotrait-Monotrait Ratio.

## 4. Result

### 4.1. Path analysis and model validation

**Fig 2:**
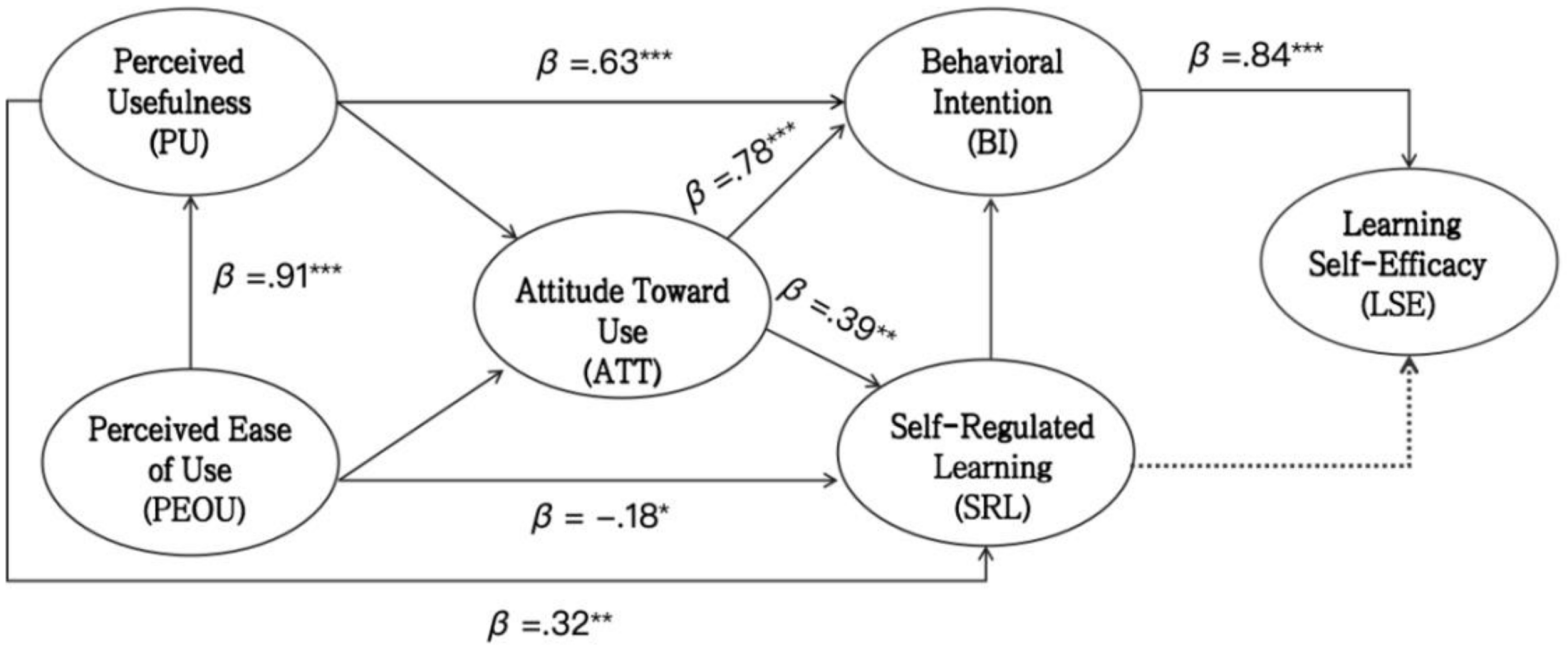
Path analysis diagram of research model. Note: *p < 0.05, **p < 0.01, **p < 0.001; --- indicates failed test.

The squares structural equation model (PLS-SEM) and the specific results are depicted in Fig 2. This figure presents the path coefficients (β) from the PLS-SEM analysis examining the relationships among perceived ease of use (PEOU), perceived usefulness (PU), attitude (ATT), behavioral intention (BI), self-regulated learning (SRL), and learning self-efficacy (LSE) in the context of the Chuanggao fitness App. The model explains a substantial portion of the variance in key endogenous constructs, with R² values of.85 for behavioral intention and.33 for learning self-efficacy. Students’ perceived ease of use of Chuanggao APP significantly and positively affects perceived usefulness (β = .91, p < .001) and perceived usefulness (β = .63, p < .001), attitude (β = .78, p < .001) showed significant positive impacts on the intention to use the applications (Apps). Self-regulated learning is greatly improved by students’ perceptions of the Chuanggao App’s usefulness (β = .32, p < .01), and there is a strong positive correlation between students’ attitudes toward using the Chuanggao App and self-regulated learning (β = .39, p < .01). Conversely, the study found that there is a significant negative correlation (β = −.18, p < .05) between perceived ease of use and self-regulated learning. Moreover, students’ intention to use the Chuanggao App substantially enhances their self-efficacy in learning (β = .84, p < .001).

Regarding the explanatory power of the model, the comprehensive explanatory power of the technology acceptance (PU, PEOU, ATT) on behavioral intention (BI) is notably high at 85% (R² = .85). Furthermore, BI explains 33% (R² = .33) of the variance in Learning Self-Efficacy (LSE), indicating that technology acceptance plays a key role as a major driver for improving learning efficiency. The specific data from this study are shown in Tables 3–5.

### 4.2. Reconciling the paradox: The negative PEOU-SRL relationship in sports education contexts

A noteworthy paradox emerged in physical education field, revealing a negative correlation between perceived ease of use (PEOU) and self-regulated learning (SRL) (β = −.18, p < .05). This finding contradicts the traditional Technology Acceptance Model (TAM) theory, which states that the ease of use of technology have a positive impact on user behavior [36], and several key factors may explain this phenomenon.

The trade-off between functional practicality and operational convenience in sports apps represented by “Chuanggao” is a key factor in forming this negative association. Students prioritize whether the apps meets their basic requirements. When apps offer more powerful practical functions, students tend to adopt more advanced self-regulated learning methods to achieve their educational goals, even if the operation process is somewhat complicated. Conversely, an overly simplistic interface could weaken students’ motivation to achieve their goals, thereby reducing their learning autonomy. Then, the conflict between the mandatory nature of task design and students’ learning autonomy plays a driving role in the negative relationship. When the task design of the mobile application (APP) is inflexible, high ease of use forces students to complete tasks passively instead of actively developing personalized exercise plans, which inhibits students’ SRL ability. As some students reported: The APP operation is too simple, which makes me too lazy to think about how to optimize the exercise plan. This shows that although high ease of use helps complete basic tasks in the short term, it may inhibit students’ learning initiative and self-regulation ability in the long run. Furthermore, technology-dependent risk also plays a crucial role in the inverse correlation between Perceived Ease of Use (PEOU) and Self-Regulated Learning (SRL). Increased reliance on technological aids could hinder students’ initiative in formulating exercise plans. Nikou and Economides(2021) [30] pointed out that the excessive user-friendliness of the Chuanggao App led to excessive overreliance on technological tools during learning and practice, thereby diminishing students’ ability to construct knowledge autonomously.

Thus, sports-related apps, like “Creative Learning,” should therefore be designed with more than just basic usability in mind. Establishing supportive yet demanding learning environments is crucial. In order to accomplish this, designers must properly balance promoting students’ learning autonomy with technological usability [45,46]. The study offers a more comprehensive and dynamic theoretical explanatory framework for Technology Acceptance Theory by creatively exposing the non-linear impact mechanism of technological usability on self-regulated learning. The discovery bridges the gap between technology, psychology, and educational practice by offering forward-looking theoretical guidance for educational technology design in addition to enhancing the meaning of Technology Acceptance Theory.

### 4.3. Dual effects and boundary extensions: Reconceptualizing TAM in sports pedagogy

The primary determinants of technology acceptance are perceived usefulness and attitude toward use, which together represent the most influential factors affecting users’ willingness to adopt a technology, accounting for 72% of the total effect. This finding implies that app developers should prioritize enhancing the functional utility of app features while cultivating users’ emotional engagement [47]. An effective sports app should not only meet users’ practical needs but also combine attractive interface design to strengthen emotional connections, thereby increasing users’ adoption intentions.

The study highlights the dual impact of psychological regulation, emphasizing the perceived usefulness and attitude towards Chuanggao App play a key role in enhancing students’ self-regulated learning (SRL) ability rather than focusing only on perceived ease of use. The inverse relationship between PEOU and SRL highlights a potential usability trap. Overly simplistic app designs may discourage students from engaging in deep learning practices, leading to poor learning outcomes. Thus, developers are advised to strike a balance between user-friendliness and functional complexity during app design and development to stimulate students’ higher-order learning processes and skill acquisition.

Regarding transformation mechanism of learning self-efficacy, the intention to use sports apps represented by Chuanggao has a significant positive influence on learning self-efficacy (β = .84, p < .01). This suggests that technology acceptance directly affect students’ confidence in their own abilities. These findings empirically support the learning by using, indicating that higher product acceptance is associated with increased cognitive engagement and self-confidence in the learning process. Accordingly, the rational use of technology can enhance students’ learning outcomes and stimulate their learning motivation and self-efficacy.

To enhance the sports app users’ intention to use, developers should focus on perceived usefulness and user attitudes while avoiding the drawbacks of overly simplified design. By optimizing functions and strengthening emotional identification, users can participate more deeply and enhance their autonomous learning ability, and ultimately achieve significant improvement in learning outcomes.

## 5. Discussion

### 5.1. Theoretical boundaries of TAM in physical education and educational technology

This study integrates self-regulated learning (SRL) and learning self-efficacy (LSE) into the technology acceptance model (TAM) to contract a three-component framework of “technology-psychology-behavior”. This framework systematically reveals the multi-dimensional driving mechanisms underlying sports app acceptance and provides a new theoretical perspective for the research on blend sports teaching.

Self-regulated learning (SRL) servers as a mediator between perceived usefulness (PU), perceived ease of use (PEOU), and behavioral intention (BI). Our structural model indicates that SRL partially mediates the influence of PU (β = .32, p < .01) and PEOU on BI, accounting for 32% and 28% of the total effect, respectively. These findings challenge the direct PU/PEOU→BI path proposed by Davis et al. [38]. Moreover, the perceived intensity of high SRL users was 1.7 times higher than that of low SRL users in both autonomous and personalized motor scenarios (β = .63 vs..37, p < .001). This observations aligns with mobile learning paradigm proposed by Nikou and Economides [30] and underscores the cognitive processes required for motor skills development. Specifically, motor skill improvement relies on ongoing strategy adjustment, positioning SRL not only as a mediator but a learning amplifier that converts technological utility into sustained engagement.

An important hub for modifying technology acceptance behavior is learning self-efficacy (LSE). The BI → LSE path (β = 0.84, p < 0.001) shows that for every 1 standard deviation increase in APP usage intention, students’ self-efficacy increases by 0.84 standard deviations, which is 105% higher than that reported by Wong et al. [48] in mathematics education (β = 0.41). This divergence may stem from the embodied cognitive characteristics of sports activities, such as the immediate progress indicators in endurance based tasks, which can provide immediate efficacy feedback, forming a positive feedback loop of “behavior-confidence-behavior”. Further analysis showed that the effect was significantly enhanced in the female group (Δβ=+0.11, p < 0.05), suggesting that technology-facilitated LSE development may partly mitigate gender differences in sports participation.

A notable departure from the conventional TAM principle the “usability-first” tenet, which is the observed negative correlation between perceived usefulness and self-regulated learning (β = −0.18, p < 0.05). The findings echoes the so-called “usability trap” documented in sports contexts [31,49], where overly simple interfaces can dampen learners’ motivation to regulate strategies. In line with Davis (1989) [38], PEOU is not universally beneficially, while in some contexts, moderately complex interfaces may better promote SRL by providing the cognitive challenge necessary for adaptive learning. Accordingly, sports app design should strike a balance between ease of use and “strategic complexity”, facilitating adaptive SRL rather than solely maximizing convenience.

Collectively, these results recast TAM from a narrow technology acceptance framework into a psycho-instructional ecosystem. Responsing to Venkatesh et al. [50], this study explores how technological features interact with learners’ psychological states to jointly shape educational outcomes by establishing SRL and LSE at the center of the framework.

### 5.2. Constructing a trinity hybrid sports teaching optimization framework

Create cognitive-affective design solutions based on perceived usefulness (PU). Developers have revolutionized sports apps into tools that enhance students’ self-regulated learning (SRL) by incorporating visualization modules rooted in physiological mechanisms, such as dynamic demonstrations and gradient target systems based on EPOC metabolic principles. These apps can effectively enhance students’ self-regulate learning abilities and stimulate learning motivation. To strengthen perceived usefulness (PU), it is recommended to incorporate course modules on high-intensity interval training (HIIT) and sports injury prevention, to help students understand the physiological mechanisms behind the functions by embedding scientific training principle explanations. Additionally, allowing participants to set personalized goals based on their physical fitness levels significantly enhances their SRL abilities.

Develop a technical-psychological synergistic support system based on the transmission path of behavioral intention (BI) and learning self-efficacy (LSE). Instructors should implement differentiated instruction for low-efficacy groups, assigning foundational tasks (e.g., completing 15 effective exercises) or challenging tasks (e.g., breaking personal best records) according to LSE levels. Encourage higher-level LSE students to autonomously design tasks to stimulate the use of innovation. The course employs blended assessment that 60% quantitative progress tracking and 40% evaluation of strategic innovation. This approach aims to overcome the usability paradox and create a more effective learning environment.

We offer an innovative context-aware authentication protocol that employs dual verification through GPS tracking and biometric identification. This approach prevents fraudulent check-ins in blended learning environments, ensuring data authenticity and mitigating risks associated with technological dependency. Concurrently, we establish a data literacy empowerment mechanism to cultivate teachers’ data diagnosis skills. This enables them to identify students’ learning weaknesses through application feedback analysis, facilitating a role of transition from “content deliverers” to “learning strategy coach.”

Overall, the study proposes a threefold strategy encompassing “cognitive reconstruction, skill-mindset integration, and ethical governance.” This comprehensive framework aims to guide the digital transformation of physical education, addressing technological innovation needs while demonstrating the practice value of theoretical research in educational practice.

### 5.3. Physical education technology acceptance: Cultural dependence and context specificity

The cultural dependency and context-specific nature of the Technology Acceptance Model (TAM) in physical education provide valuable insights for advancing theoretical frameworks. Research indicates that Self-Regulated Learning (SRL) had no significant impact on Behavioral Intention (BI) deviated from the findings of Shih et al. [51] in theoretical online courses. This discrepancy may stem from fundamental differences in instructional settings and cultural environments. Shih et al.(2018) [51] focused on theoretical course closely aligned with self-regulate learning, whereas tasks in physical education are predominantly practice-based, potentially reducing students’ autonomy to employ SRL strategies. The Chuanggao app ex-amplifies current limitations in sports applications, highlighting issues such as overemphasis on task completion, lack of like goal setting features SRL support, and mismatches between student capabilities and behaviors.

More significantly, the study found a negatively connected with self-regulated learning (SRL), posing a significant challenge to Davis et al.(1989) [38] traditional TAM theory. Han (2021) [50] and Al-Adwan et al. (2023) [31] have both confirmed that overly simplified user interfaces may inhibit learners’ motivation to adjust strategies. A “autonomy threshold” may be formed within a particular cultural context, and in the collectivist educational context of East Asia, a moderate increase in system complexity may actually be more conducive to stimulating individuals’ need for autonomous regulation. Wong et al. (2022) [25] found that learning self-efficacy (LSE) significantly positively influenced technology acceptance in mathematics learning contexts (β = .41, p < .01). However, the findings from this study in education (β = .84, p < .001) significantly exceeded this results. This discrepancy may stem from the embodied nature of physical learning, where players perceptions of their own abilities are more readily stimulated by the instant feedback of increased physical fitness. Sports app design, like Chuanggao, should prioritize user self-efficacy by incorporating data visualization, personalized progress tracking, and competitive rankings to bolster users’ self-efficacy, thereby fostering sustained engagement.

Specifically, the study reveals the mechanisms through which cultural factors influence technology acceptance, proving a nuanced interpretations of TAM applications in physical education contexts and highlighting the interplay between technology, psychology, and cultural factors.

### 5.4. Research limitations and future directions

Research on the technology acceptance model has made progress but still faces limitations. Firstly, the study external validity and generalizability may be considerably diminished if the sample selection process is limited to a single institution. Students learning behaviors and experiences may be affected by disciplinary characteristics, cultural background, urban-rural differences, and institutional differences. Future research should expand sample scope to include a more diverse population to increase the generalizability and explanatory power of the findings. Secondly, cross-sectional research designs struggle to reveal the dynamic interactions process and causal mechanisms between learning self-efficacy (LSE) and self-regulated learning (SRL). Therefore, it is recommended that future studies adopt a longitudinal tracking design to systematically explore the interaction and evolution of LSE and SRL through long-term tracking studies. Furthermore, the study cross-cultural research perspective limits its theoretical depth. Comparative studies should be conducted to clarify the applicability and variation characteristics of technology acceptance models across different cultures. Lastly, this study focuses on the physical education context, providing a theoretical and methodological foundation for subsequent research. The goal is to enhance the technology acceptance model within the education sector through continuous theoretical advancements and empirical research.

## 6. Conclusion and recommendations

### 6.1. Conclusion

#### 6.1.1. Driving mechanisms of technology acceptance paths.

Perceived Usefulness (PU) plays a key factor in consumers’ intention to use sports apps in the technology acceptance model. The study demonstrates that PU has a substantial and favorable impact on intention to use (β = .63, p < .001), supporting the core hypothesis of RQ1, which states that students are more concerned about whether the APP would improve them with their physical health or courses points. Furthermore, the emotional reinforcement effect of usage attitude drives users’ intention to use (β = 0.78, p < .001), indicating that students’ positive emotional identification with the APP (e.g.,“it is fun to use the APP “) is a key emotional bond for technology acceptance.

#### 6.1.2. Bidirectional effects of psychological regulation mechanisms.

The study found that the mediating role of SRL, namely PU and ATT, positively affects SRL (β = 0.32**, β = 0.39**), indirectly promotes college students’ intention to use sports apps, and answers RQ2, which states that students’ capacity to self-regulate their learning is necessary for technology adoption. The substantial inverse relationship (β = −0.18, p < .05) between self-regulated learning and perceived ease of use points to a “ease of use trap,” in which a simplistic design might hinder students’ motivation to adjust strategies.

#### 6.1.3. Transformational path of learning self-efficacy.

Intention to use sports apps have a strong driving effect on learning self-efficacy (LSE) (β = 0.84, p < .001), indicating that technology acceptance behavior could be directly transformed into students’ confidence in their own learning abilities, thus supporting the hypothesis of learning by use in RQ3.

### 6.2. Recommendations

#### 6.2.1. Optimize the functional design of the sports app.

Sports applications should be designed with the principles of hierarchy and science. On the one hand, a hierarchical structure of modules is implemented. The basic module includes standardized tasks such as running three times a week to ensure standardized teaching and assessment; the advanced module allows customized goal setting to increase student engagement in the application. Through the built-in sports physiology knowledge base, such as interval running and cardiopulmonary function improvement mechanisms, etc. While improving students’ learning cognitive level, a dynamic feedback system is introduced to strengthen process monitoring and timely adjustment, thus activating the closed-loop mechanism of monitoring – assessment – correction in the SRL path.

#### 6.2.2. Innovative teaching design strategy.

The teaching design is closely integrated with student motivation and individual differences and supports the methods of “task differentiation” and “assessment diversity.” Students were provided activities based on their level of self-efficacy. The low-LSE group focuses on completing systematic tasks to build basic confidence, and the high-LSE student group guides them to set challenging goals to stimulate advanced motivation. With the use of a sports app, teachers gather training data and are alerted to any specific training deficiencies, recognizing the exact intervention of using data to promote teaching”. Additionally, we should broaden the evaluation dimension and add strategy application indicators on the basis of completing the specified number of runs and distances to evaluate students’ use of learning strategies, thereby promoting the systematic cultivation of SRL abilities.

#### 6.2.3. Strengthen the management mechanism.

Technical means are key to high-quality instruction. The speed anomaly alert feature could help teachers review suspicious data, and integrating GPS tracking and bio-metrics enhances data validity. To better meet the needs of the teaching profession and enhance the fit between the apps and the course objectives, relevant departments of the school should focus on improving teachers’ the digital teaching skills and establish a Technology-Teaching Collaboration Working Group to promote direct communication between educators and sports app developers.

#### 6.2.4. Promoting psychological adaptation and learning motivation stimulation.

Students’ long-term propensity to utilize the sports app would be greatly increased if it were well-connected to their psychological processes. Integrating a stage accomplishment system, like advanced medals for completing three consecutive weeks of standardized exercises, into the APP module is proposed to enhance self-efficacy through immediate feedback. For students’ learning motivation, social interaction modules are added, such as leader-boards, challenges, and comment-sharing functions, to enhance students’ usage attitudes and participation stickiness. Studies have shown that moderate rivalry and outside incentives have a big impact on students’ collective culture, it seems sensible to include them on the platform.

Overall, this study collaboratively improves the technical support and educational effectiveness of blended physical education by optimizing the technical structure of sports apps, reconstructing teaching design, strengthening management mechanisms, and paying attention to students’ psychological adaptation, and it provides empirical evidence and a systematic path for the in-formalization transformation of physical education in colleges and universities. Future studies could also further promote the innovative development of blended physical education by expanding sample diversity, deepening longitudinal analysis, and exploring cultural differences.

## Supporting information

S1 File Valid data. (XLSX)
